# EHMTI-0283. Predictors of early response to onabotulinumtoxin type a in chronic migraine

**DOI:** 10.1186/1129-2377-15-S1-G31

**Published:** 2014-09-18

**Authors:** N Mas-Sala, M Quintana, J Alvarez-Sabin, P Pozo-Rosich

**Affiliations:** 1 Neurology, Hospital Universitari Vall d'Hebron, Barcelona, Spain

## 

To analyse predictors of early response after two treatment cycles with Onabotulinumtoxin type A (OnabotA) in patients with chronic migraine (CM).

Thirty-one patients (24 women, 7 men) with IHS criteria for CM and an inadequate response or intolerance to other oral preventatives were included. Data regarding the frequency and intensity of headache attacks, analgesic use and migraine disability assessment scale (MIDAS) before and after OnabotA treatment was collected; as well as, headache location and the presence of allodynia or bruxism. The current oral preventive therapy was continued in all patients.

Good responders were patients who after treatment improved in all of these four categories: headache frequency (CM to low-frequency episodic migraine), headpain intensity (reduction of >75%), analgesic/triptan use (reduction to a use of two days or less per week), and disability (from severe to a mild disability measured using the MIDAS scale). Partial responders were those who only improved in two of the four categories.

Patients were classified into 3 categories: good responders (58.1%), partial responders (29%), and non-responders (12.9%). The age (younger subjects) and the presence of a shorter migraine history are the two indicators of a better response to the treatment (p=0.04; p=0.04 respectively). Unilateral headache, presence of allodynia and/or bruxism appears not to be predictors of response to OnabotA in our study.

Age and the years of migraine history are predictors of early response to OnabotA in our experience. The younger the patient and less years of migraine chronification are good predictors of early response.

